# Cell Senescence in Myxoid/Round Cell Liposarcoma

**DOI:** 10.1155/2014/208786

**Published:** 2014-06-24

**Authors:** Christina Kåbjörn Gustafsson, Anders Ståhlberg, Katarina Engtröm, Anna Danielsson, Ingela Turesson, Pierre Åman

**Affiliations:** ^1^Sahlgrenska Cancer Center, Department of Pathology, Institute of Biomedicine, University of Gothenburg, Box 425, 40530 Gothenburg, Sweden; ^2^Department of Oncology, Institute of Medical Sciences, University of Gothenburg, Blå stråket 2, 41345 Gothenburg, Sweden; ^3^Department of Oncology, Institute of Medical Sciences, Uppsala University, 75185 Uppsala, Sweden

## Abstract

Myxoid/round cell liposarcoma (MLS/RCLS) is the second most common liposarcoma type and characterized by the fusion oncogenes *FUS-DDIT3* or *EWSR1-DDIT3*. Previous analysis of cell cycle regulatory proteins revealed a prominent expression of G1-cyclins, cyclin dependent kinases, and their inhibitors but very few cells progressing through the G1/S boundary. Here, we extend the investigation to proteins involved in cell senescence in an immunohistochemistry based study of 17 MLS/RCLS cases. Large subpopulations of tumor cells expressed the RBL2 pocket protein and senescence associated heterochromatin 1*γ* and IL8 receptor *β*. We conclude that MLS/RCLS tissues contain major populations of senescent tumor cells and this may explain the slow growth rate of this tumor type.

## 1. Introduction

Liposarcoma is the most common soft tissue tumor in humans and myxoid/round cell liposarcoma (MLS/RCLS) is the second most common liposarcoma type. In a majority of the cases MLS/RCLS develops in large muscles, most often in the thigh [1]. MLS/RCLS tissue is composed of round to oval shaped mesenchymal cells and a variable number of lipoblasts, set in a myxoid matrix with a fine piped capillary network. Most cases of MLS tumors are relatively slow growing but 10–15% show a hyper cellular round cell morphology (RCLS) with less myxoid component that is associated with an unfavourable prognosis [1].

The fusion oncogenes* FUS-DDIT3* or* EWSR1-DDIT3* are present in almost all cases of MLS/RCLS. They result from* t*(12; 16) or* t*(12; 22) chromosome translocation and have causative roles in development of MLS/RCLS [2–5].* FUS-DDIT3* and* EWSR1-DDIT3* encode abnormal transcription factors that deregulate the expression of target genes [6–8]. For example, we previously showed that* FUS-DDIT3* directly induces production of IL6 and IL8 by binding to the* IL6* and* IL8* promoters [9, 10].

A recent study reports that most MLS/RCLS cases also carry TERT promoter mutations, suggesting an increased TERT activity and extended cellular life span [11]. The RCLS variant is also associated with additional mutations, most commonly in PIK3CA [12].

Previous studies of cell cycle regulating proteins in MLS/RCLS revealed a prominent expression of growth promoting G1 cyclins and cyclin dependent kinases (CDK), coexpressed with cyclin dependent kinase inhibitors P16, P19, and P27 (also known as CDKN2A, CDKN2D, and CDKN1B, resp.) [13]. This pattern suggested that the growth promoting cyclin/CDK activity was counteracted by CDK inhibitors and this could explain the low frequency of proliferating cells identified by Ki67 and cyclin A staining (typically less than 8 and 4 percent, resp.) [13].

Recent results from investigations of senescent cells suggest that they express G1 cyclins, CDKs, and their inhibitors as well as a typical expression signature of cytokines and their receptors [14–17]. These new data and concepts provide a possible explanation for the expression patterns observed in MLS/RCLS and prompted a renewed investigation with focus on senescence markers. In the present study, we have employed immunohistochemistry (IHC) and analysed a cohort of 17 MLS/RCLS cases for expression of proteins associated with growth control and senescence. The results suggest that substantial proportions of MLS/RCLS tumor cells are senescent.

## 2. Materials and Methods

### 2.1. Tissue Samples

The clinicopathological characteristics of all tumors are presented in Table 1. Formalin-fixed tissues taken at surgery from previously untreated cases with MLS/RCLS were embedded in paraffin using routine procedures and stored at room temperature. Use of tissue samples for this study was examined and approved by the Ethical Board associated with the University of Gothenburg. All selected MLS cases were examined by two clinical pathologists specialized in soft tissue tumors and by the first author, also a clinical pathologist.

### 2.2. Cell Cultures

Human foreskin derived fibroblasts (passage 8) were cultured in RPMI1640 with 5% fetal calf serum. Immunofluorescence studies were made 5 days after a 10 Gy dose of X-ray radiation. MLS cell lines 402-91, 1765-92, and 2645-94 and HT1080 clones [4, 18, 19] were cultured in RPMI1640 with GlutaMAX and 8% fetal bovine serum, 100 U/mL penicillin, and 100 *μ*g/mL streptomycin. All media and supplements were obtained from Life Technologies. All cells were maintained at 37°C with air containing 5% CO_2_.

### 2.3. IL-6 Dependence Assay

MLS cell lines 402-91, 1765-92, and 2645-94 were seeded to 96 well microtiter plates at 2000 cells/well and allowed to settle for 8 hours. The culture medium was replaced with medium supplemented with 3% fetal bovine serum, with and without 1 unit/mL of recombinant IL6 and with or without 0.5 *μ*g/mL of monoclonal IL6 antibody mAB 206 (R&D systems). Trypan blue excluding cells were counted in an inverted microscope after 48 hours of incubation. An Epstein-Barr virus immortalized lymphoblastoid cell line was included as positively responding control [20].

### 2.4. Immunohistochemistry and Immunofluorescence Microscopy

Series of 5 *μ*m tissue sections were cut from each biopsy, deparaffinised, rehydrated, boiled in microwave oven for 10 minutes for epitope retrievement, and stained with the antibodies listed in Table 2. Bound antibodies were visualized using the LSAB secondary antibody streptavidin biotin peroxidase system (DAKO). Stained sections were examined on a light microscope.

For immunofluorescence analysis, cultures of human fibroblasts were washed twice with PBS and fixed in 4% paraformaldehyde in PBS. After two more washes in PBS, the slides were mounted in an antifade mount containing the DNA binding dye DAPI (4,6-diamidino-2-phenylindole dihydrochloride) (Olink Bioscience) and examined on a fluorescence microscope. Several antibodies were tested for each antigen and evaluated by IHC staining of tissue sections containing published positive and negative cell populations. The selected primary antibodies were also tested by western blot analysis of MLS cell lines and other reference cell lines as described elsewhere [13]. Irradiated fibroblasts were used as a control for the senescence markers used (Table 3). Detection of RB1 expression worked satisfactorily only with the antibody and protocol detailed in Table 2. Evaluation of IHC stains was made by Christina Kåbjörn, specialist pathologist, and Pierre Åman.

### 2.5. Flow Cytometric Analysis of Cell Cycle Phase Distribution

Paraffin-embedded, formalin-fixed tissues were dewaxed, and single-cell suspensions were prepared and labeled with propidium iodide (Life Technology, catalogue number P3566) according to the providers protocol and as previously described [21]. Flow cytometric analysis of DNA content was evaluated using the FACS calibur system (BD Biosciences). The ModFit LT software (Verity Software House) was used for analysis and peak detection to identify distributions of G1, S, and G2 cells.

### 2.6. FISH Analysis

Interphase FISH analysis of FUS-DDIT3 and EWSR1-DDIT3 rearrangements [22] was performed on formalin-fixed 1–4 *μ*m paraffin tissue sections. Three break-apart probes, DDIT3, FUS,and EWSR1 (Vysis Inc.), were used according to protocols supplied by the manufacturer. Nuclei were counterstained with 10 *μ*L 4′,6′,-diamidino-2′-phenylindole dihydrochloride (DAPI). The sections were analyzed and reanalyzed by two independent reviewers. At least 100 nuclei per section were scored. The interpretation of intact, fusion, and split signals was based on guidelines recommended by the manufacturer and from other clinical laboratories using this method.

## 3. Results

Irradiated fibroblasts were used as a control for the senescence markers and antibodies used. Our data showed a very strong expression of all investigated senescence associated markers in irradiated cells compared to control cultured cells (Table 3).

**Figure 1:**
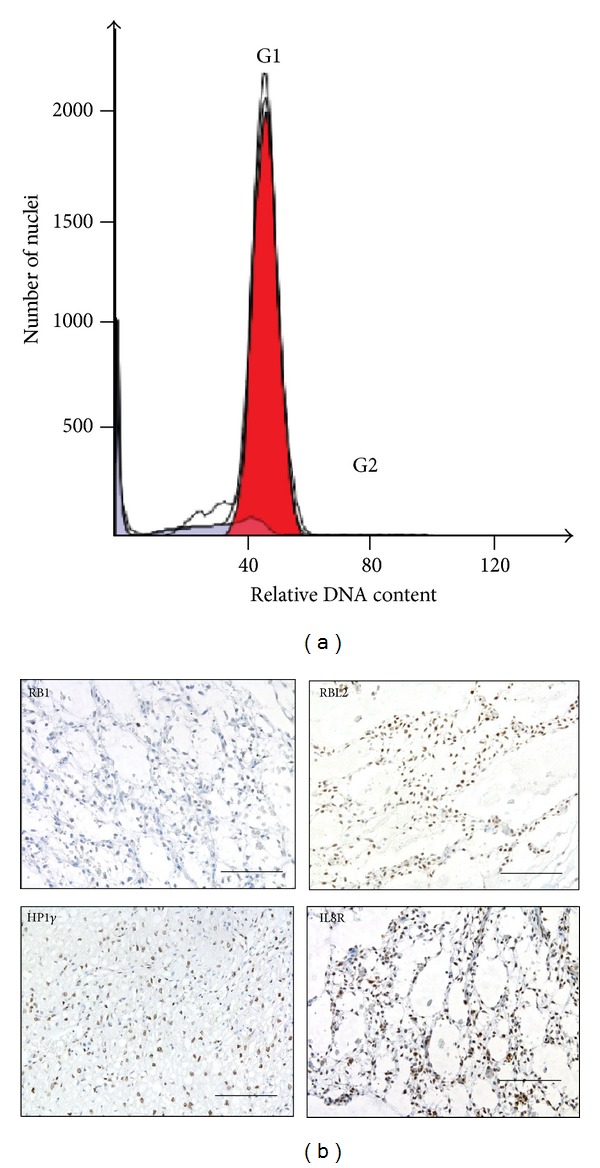
(a) Flow cytometry histogram of cell nuclei extracted from paraffin embedded MLS/RCLS tumor tissue (Cases 8) showing more than 95% of the cells in the G1 phase of the cell cycle (red). (b) Immunohistochemistry analysis of RB1, RBL2, HP1*γ*, and IL8R in MLS/RCLS tumor tissues. Brown staining shows reactivity with the specific antibodies. Bars are 100 *μ*m.

Our Ki67 IHC staining results (Table 1) and previous data [13] showed that most MLS cases contain very few Ki 67 positive cells, typically less than 4%, but some RCLS tissues contained smaller tissue regions with up to 8% positive cells. A previous investigation also showed only few cells expressing the S-G2 phase specific cyclin A, suggesting that a majority of the cells are arrested in the G1 phase [13]. Here, our flow cytometry analysis of cell nuclei from two human MLS/RCLS tissues showed 94% and 96% G1-phase cells, respectively (Figure 1), supporting our conclusion that most MLS/RCLS tumor cells were arrested in the G1 phase of the cell cycle.

IHC analysis of pocket proteins showed that 9–94% of the cells expressed the proliferation associated RB1 and 0–93% expressed the rest of phase protein RBL2. It is thus obvious that there are overlaps in expression of the RB1 and RBL2 proteins.

**Figure 2:**
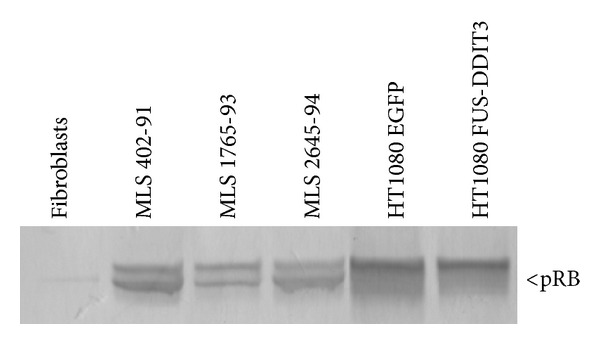
Western blot analysis of RB1 expression in cultured normal human fibroblasts, MLS cell lines, and FUS-DDIT3 or EGFP transfected HT1080 cells.

Our IHC analysis showed only weak signals for the RB1 protein in MLS tissues compared to control tissue samples from other tumor types. This prompted a further analysis of RB1 protein expression in MLS cells. Western blot analysis showed a strong expression of a normal sized RB1 protein in all three investigated MLS derived cell lines (Figure 2). Forced expression of FUS-DDIT3 gave no effects on RB1 expression in the human HT1080 fibrosarcoma cell line (Figure 2).

Between 14 and 76% of the tumor cells stained positive for heterochromatin protein (HP1*γ*) (Table 1 and Figure 1). Together with the pocket protein results, these data suggest that a large proportion of the MLS/RCLS cells are arrested in the G1 phase and that the tumors contain a substantial fraction of senescent cells.

Cell senescence is associated with a distinct cytokine and cytokine receptor expression signature and FUS-DDIT3 is known to induce IL6 and IL8 expression in the tumor cells. IHC analysis of MLS/RCLS tissues showed that the senescence associated IL8 receptor beta (also known as CXCR2) was expressed in 50–91% of the tumor cells (Table 1 and Figure 1). We tested MLS/RCLS tumor cell lines for in vitro growth and survival dependence on IL6, but no effects of treatment with IL6 or IL6 blocking antibodies were detected (data not shown).

## 4. Discussion

Our previous analysis of cell cycle regulator expression in MLS/RCLS suggested that a majority of the tumor cells were arrested in the G1 phase of the cell cycle [13]. Only a few percent of the cells escape this arrest as detected by low numbers of Ki67 and cyclin A positive cells. This conclusion is supported here by the present Ki67 IHC analysis of 17 MLS/RCLS cases and by flow cytometry analysis of two cases.

The Ki67 and cytometry analysis was complemented by an IHC based investigation of the pocket proteins RB1 and RBL2 (also known as P105 and P130). RB1 and RBL2 are important control hubs for cell cycle regulation and proliferation driving transcription factors. RB1 is expressed in proliferating cells and is also necessary for induction of senescence, while RBL2 is expressed in resting and senescent cells. Consequently, the levels of RB1 decrease and the levels of RBL2 increase, as cells enter a nonproliferative or senescent cell state [23–27]. Our IHC results for RB1 and RBL2 support the previous conclusion that large subpopulations of tumor cells are in a resting state.

IHC staining of RB1 in MLS/RCLS tissues gave generally weak signals calling for further analysis. Six different RB1 specific antibodies and various staining/antigen retrieving conditions were tested (data not shown). Compared to the staining intensities for RBL2 and HP1*γ*, the signal was always found weaker in MLS tumor tissues and RB1 staining was also weaker in MLS tissues compared to reference tumor tissues of other entities. Analysis of MLS/RCLS derived cell lines showed RB1 protein expression in level with other tumor cell lines and much stronger than the expression in cultured normal fibroblasts (Figure 2). Comparison of HT1080 fibrosarcoma cells with and without the* FUS-DDIT3* showed that the fusion protein has no direct effect on RB1 expression (Figure 2). Taken together, our data suggest that MLS/RCLS cells are capable of normal expression of RB1. The fading RB1 expression in MLS/RCLS may thus result from a normal downregulation in connection with growth cessation of many tumor cells.

Some senescent cell types are characterized by nuclear heterochromatin foci that can be visualized by DNA-stains and the increased expression of heterochromatin protein 1 gamma (HP1*γ*) [28, 29]. The IHC analysis of HP1*γ* in MLS/RCLS tissues showed a heterogeneous pattern with large numbers of strongly stained cells. This suggests that large subpopulations of the tumor cells are senescent with expanded heterochromatin formation and thus they may be permanently excluded from further proliferation.

**Figure 3:**
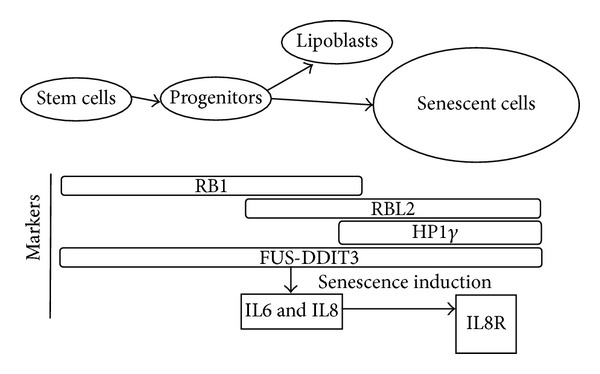
Schematic presentation of tumor populations and factors involved in senescence. A small population of proliferating cells arises from a hypothetical tumor stem cell population. Most of these cells enter senescence and a few percent differentiates into lipoblasts. RB1 and RBL2 proteins are central factors involved in growth regulation and entry/maintenance of cell senescence. RB1 is expressed in proliferating cells and is also necessary for differentiation and senescence. RBL2 is expressed in resting and senescent cells. Increased HP1*γ* expression is typical for senescent cells. IL6 and IL8 are produced by the tumor cells and IL8 may bind to the IL8*β* receptor expressed by senescent tumor cells.

FUS-DDIT3 binds the promoter regions of the* IL8* and* IL6* encoding genes leading to expression of these genes [9, 10]. IL6 is reported as an autocrine growth or survival factor in several tumor types [30–33]. Our results suggested, however, that IL6 is not a growth/survival factor for MLS/RCLS cells. More recently IL6 and IL8 were reported to be parts of a cytokine expression profile (IL6 and IL8) that is typical for senescent cells [34–36]. Instead of acting as growth factors, IL6 together with IL8 may thus be part of a senescence mechanism in MLS/RCLS. Our data showing IL8 receptor expression in many of the tumor cells is in line with this interpretation. The IL8 receptor beta expression also indicates a possible senescence associated IL8 autocrine activity as the tumor cells also are producing IL8 [7]. A schematic presentation of the investigated senescence associated factors in MLS/RCLS is shown in Figure 3.

Our hypothesis that major subpopulations of MLS/RCLS cells are senescent may seem contradictory to the recent report that MLS/RCLS tumors carry TERT promoter mutations. Such mutations may indicate an increased TERT activity and immortalization of the tumor cells [11]. The large numbers of senescent cells in MLS/RCLS tumors may, however, be caused by oncogene induced stress responses resulting in senescence. This hypothesis is supported by our previous in vitro experiments with FUS-DDIT3 transfection into various cell types. Forced FUS-DDIT3 expression caused cell death and senescence in most cell types and only very few cells in permissive cell lines maintained proliferative capacity [18]. Surviving FUS-DDIT3 transfected cells also had a slower in vivo and in vitro growth rate compared to wild-type cells [18]. Oncogenic stress induced senescence has been reported for many oncogenes and is thought to serve as a major barrier against tumor development in vivo [14–17]. This mechanism may also explain the observed low growth rate and abundance of senescent cells in MLS/RCLS tumors.

Ten to fifteen percent of MLS/RCLS cases show round cell morphology and this is associated with increased growth rate and unfavorable prognosis. Inspection of our limited cohort of cases failed to detect any correlation between the RCLS morphology and expression data of the analyzed markers. A possible association between RCLS morphology and number of senescent cells has to be tested in a larger tumor material.

Irradiated fibroblasts showed a very strong expression of all investigated senescence associated markers. In a clinical study, we have previously reported that irradiation of MLS/RCLS tumors results in transformation to a lipoma like morphology with highly differentiated adipocyte like cells [37]. Further investigations of irradiated MLS/RCLS tumors will show if this treatment causes maturation of senescent cells into adipocyte like cells. Such effects have been reported in other tumor types [38].

We have investigated 17 MLS/RCLS tumors for expression of senescence associated proteins. The results suggest that large subpopulations of tumor cells are in a senescent cell state characterized by expression of HP1*γ*, RBL2, and senescence associated cytokines and a cytokine receptor. The presence of large numbers of senescent cells may explain the observed slow growth rate of this sarcoma type.

## Figures and Tables

**Table 1:** Cases and immunohistochemistry data.

Case	Age	Site		RB1^1^ (%)	HP1*γ* ^1^ (%)	RBL2^1^ (%)	IL8R^1^ (%)	KI67^1^ (%)	FUS/DDIT3 rearrangement^2^	Histological classification
1	42	im	Hip	35	50	79	55	nd	+	MLS
2	45	im	Thigh	23	66	77	86	<1	+	MLS
3	37	im	Thigh	73	50	69	69	4	+	MLS
4	36	im	Thigh	60	56	55	86	<1	+	MLS
5	80	im	Thigh	62	60	93	91	2	+	MLS
6	34	im	Thigh	58	44	91	79	3	+	MLS
7	56	im	Thigh	72	47	76	64	<1	+	MLS
8	46	sc	Thigh	65	51	85	86	1	+	MLS
9	73	im	Lower leg	75	49	83	83	<1	nd	MLS
10	45	other	Peritoneal	73	54	80	80	3	nd	MLS
11	49	sc	Thorax met	94	56	93	90	nd	+	MLS
12	33	other	Peritoneal	25	76	87	50	<1	+	MLS/RCLS
13	37	im	Leg	9	14	69	62	nd	nd	MLS/RCLS
14	38	im	Thigh	81	47	68	85	6	+	MLS/RCLS
15	76	sc	Back	73	50	82	54	<1	+	MLS/RCLS
16	46	im	Hip	85	45	0	74	8	+	RCLS
17	19	im	Shoulder	77	51	78	79	7	nd	RCLS

^1^Percent cells expressing respective protein.

^2^ *FUS-DDIT3* rearrangement status as determined by FISH. ^+^Indicates presence of *FUS-DDIT3* rearrangements. nd: not determined due to technical problems or lack of material.

im: intramuscular; sc: subcutaneous; nd: not determined; met: metastasis.

**Table 2:** Antibodies used for immunohistochemistry and immunofluorescence.

Antigen	Antibody	Dilution	Retrievement
IL8R*β*	SC-23811	1 : 50	PH6
RBL2	SC-53641	1 : 50	PH9
RB1	BD #5544136	1 : 20	PH9
TP53	Calbio.OP43A	1 : 100	
CCNA	NM MS1061-S1	1 : 15	
P21	Millipore OP64	1 : 25	
P15	SC-56327	1 : 20	
HP1*γ*	Millipore 05-690	1 : 100	PH6
KI67	Dako IR-626	1 : 1	PH6

SC: SantaCruz Biotechnology; BD: Becton Dickinson and Company; Calbio: calbiochem; NM: neomarker; epitope retrievement was performed at indicated PH by boiling for 5 minutes.

**Table 3:** Senescence markers in irradiated fibroblasts.

Marker	Control	Irradiated
IL8R	Negative	100^++^
RBL2	8, 12 (mitotic)^++^	94, 96^+^
RB1	100^++^	30, 33^+^
P53	Negative	100^++^
CCNA	17, 20^++^	Negative
P21	Negative	100^+++^
P15	Negative	100^+^
HP1*γ*	<1^++^	100^+++^

Percentage positive cells for respective marker are shown. ^+,++,+++^ indicate weak, medium, and strong expression, respectively. Mitotic: expression seen only in mitotic cells. Results from two experiments are shown. Two values are shown when there was variation between the experiments.
